# Structural, magnetic and magnetocaloric properties of 0.75La_0.6_Ca_0.4_MnO_3_/0.25La_0.6_Sr_0.4_MnO_3_ nanocomposite manganite

**DOI:** 10.1039/c8ra05230a

**Published:** 2018-08-13

**Authors:** M. Jeddi, H. Gharsallah, M. Bekri, E. Dhahri, E. K. Hlil

**Affiliations:** Laboratoire de Physique Appliquée, Faculté des Sciences, Université de Sfax B. P. 1171 3000 Sfax Tunisia marwajeddi@gmail.com; Institut Préparatoire aux Études d'Ingénieur de Sfax, Université de Sfax B. P. 1172 3018 Sfax Tunisia; Physics Department, Rabigh College of Science and Art, King Abdulaziz University PO Box 344, Rabigh 21911 Saudi Arabia; Institut Néel, CNRS Université J. Fourier B. P. 166 38042 Grenoble France

## Abstract

The present study involves an investigation of structural, magnetic and magnetocaloric effect (MCE) properties of 0.75La_0.6_Ca_0.4_MnO_3_/0.25La_0.6_Sr_0.4_MnO_3_ composite material. Crystal structure analysis is performed by using Rietveld refinement of the X-ray diffraction patterns. The studied composite exhibits two structural phases; the rhombohedral and the orthorhombic structures corresponding to the mother compounds; La_0.6_Ca_0.4_MnO_3_ and La_0.6_Sr_0.4_MnO_3_, respectively. The scanning electron microscopy micrographs support our findings. Magnetic measurements as a function of temperature of the composite display two successive second order magnetic phase transitions at 255 and 365 K associated to both mother compounds. Therefore, a broadening of the magnetic entropy change peak is noted. A better relative cooling power (RCP) value of 360 J kg^−1^ compared to those observed in mother compounds is obtained at *μ*_0_*H* = 5 T, making of this material considered as a suitable candidate for magnetic refrigeration applications near room temperature. A consistent agreement between experimental results and numerical calculations based on the rule of mixtures has been shown. The theoretical modeling of the MCE using Landau theory reveals an acceptable concordance with experimental data indicating the importance of magnetoelastic coupling and electron interaction in the MCE properties of manganite systems. The field dependence of the magnetic entropy change is applied to study the critical behavior. Our results go in tandem with the values corresponding to the mean field model. The spontaneous magnetization values determined using the magnetic entropy change (Δ*S*_M_*vs. M*^2^) are in good agreement with those found from the classical extrapolation of Arrott curves (*μ*_0_*H*/*M vs. M*^2^).

## Abstract 2

The present study involves an investigation of structural, magnetic and magnetocaloric effect (MCE) properties of a 0.75La_0.6_Ca_0.4_MnO_3_/0.25La_0.6_Sr_0.4_MnO_3_ composite material.

## Graphical abstract

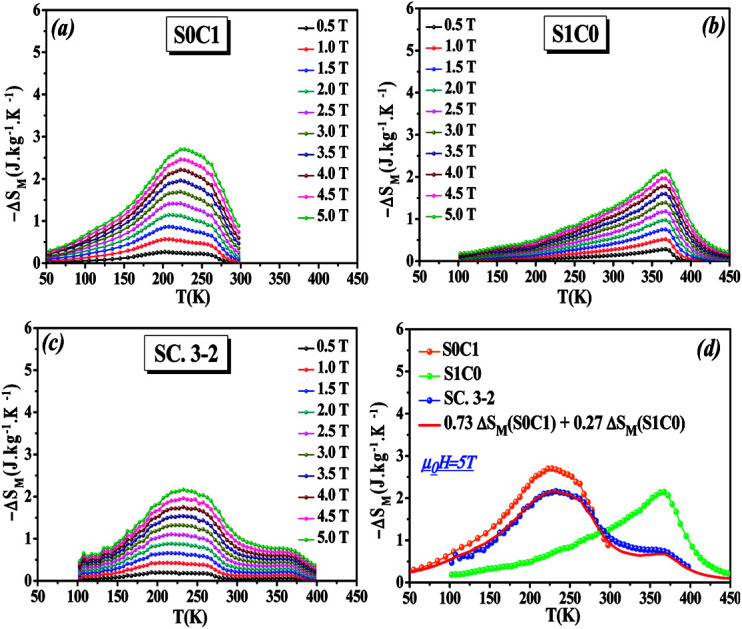

## 1. Introduction

Of all the inventions made in the last few decades, refrigeration technology has drawn a great deal of attention from the scientific research point of view. Almost of all cooling applications are mainly based on conventional gas compression/expansion. The use of gases such as chlorofluorocarbons (CFC) and hydrochlorofluorocarbons (HCFC) causes irreparable damage to our living environment.^1^ In a drive to fight the adverse effect of hazardous gases, it becomes necessary to establish a new type refrigeration technology that it is environmentally friendly and energy efficient.

Magnetic refrigeration (MR) near room temperature based on the magnetocaloric effect (MCE) has received an increasing attention owing to its several economic, ecological and energetic benefits.^1–3^ The MCE can be defined as an intrinsic property of magnetic materials. It is characterized by the temperature change (Δ*T*_ad_) in an adiabatic process and by the entropy change (Δ*S*_iso_) in an isothermal process originating from the application and removal of an external magnetic field. The major challenge of research in this field is to find out materials presenting optimal magnetocaloric properties.^4–6^ Gadolinium and some of its alloys such as Gd_5_(Ge_1−*x*_Si_*x*_)_4_ have been considered among the most active magnetic refrigerants at room temperature.^7^ Heusler alloys such as FeMnP_1−*x*_As_*x*_ or LaFe_13−*x*_Si_*x*_ have been also explored with a view to optimize magnetocaloric properties for potential applications in refrigeration.^8^ However, it was found that all these materials may exhibit numerous drawbacks such as large thermal and field hysteresis, expensive production cost, hard preparation, easy oxidation, *etc.*, which is not beneficial for the actual magnetic refrigerant application.^9,10^ Recently, large values of MCE are observed in the perovskite manganese oxides of formula (R_1−*x*_M_*x*_)MnO_3_ (where R is a trivalent rare earth ion and M is a divalent alkali earth ion).^10,11^ With large magnetic entropy change, small thermal and magnetic hysteresis, and relatively low cost,^12^ manganites have been the subject of intensive research activities for several years as the most promoter materials for refrigeration. These materials are given a particular attention from scientific community not only for its dynamic ability for uses in device applications^13–15^ but also for its impressive physical properties.^16–20^

Nowadays, researches are focusing on how to enhance the MCE values of perovskite manganites and create new material system with significant magnetocaloric properties in a wide temperature range around room temperature.^21–26^ Manganites may be promising candidates to satisfy this requirement because the transition temperature can be easily tuned by element substitution,^27^ calcination temperature,^28^ particle size,^29^ and pressure.^30^ However, the new trends have been concentrating on studying the MCE properties of manganite composite.^31–33^

The Ericsson cycle, which is one of the most basic cycles of magnetic cooling, has been proposed as the optimal process for room temperature magnetic refrigeration.^34^ In this process (two isothermal and two isomagnetic field processes), a nearly constant high magnetic entropy change (Δ*S*) over a wide temperature span is required. The efficiency of the Ericsson cycle is maximized with a constant temperature dependence of the isothermal magnetic entropy change of the magnetic samples used as working substance over the operating temperature range. In addition to the large Δ*S*, the magnetic refrigeration material needs a large value of the relative cooling power (RCP). The RCP is used as a figure of merit to measure the cooling efficiency and to characterize the magnetocaloric material when considering an ideal thermodynamic cycle.^35^ Therefore, the key issue for a magnetic refrigeration material is increasing RCP with keeping a large Δ*S*. From practical point of view, it is necessary to find a promising way to broaden the magnetic entropy curves Δ*S*(*T*) in order to increase the full width at half-maximum (δ*T*_FWHM_) and obtain the enhancement of RCP. This condition is difficult to be accomplished by a single material. To prevail over this limitation, composite materials formed by associating several magnetocaloric materials with close transition temperatures, have been extensively suggested in the literature. MCE compounds that undergo multiple successive magnetic phase transitions were found to present magnetic multi-phases that makes it possible the broadening of the Δ*S* curves with a concomitant improvement in the relative cooling power. Composite materials are obtained by assembling at least two ferromagnetic compounds in a certain weight fraction with an appropriate Curie temperature and similar values of the magnitude of the magnetic entropy change |Δ*S*^max^_M_|.

Motivated by these considerations, the purpose of this paper is to investigate the magnetocaloric properties of a composite system consisting of two perovskite manganites vividly studied La_0.6_Ca_0.4_MnO_3_ and La_0.6_Sr_0.4_MnO_3_ ^36–40^ whose Curie temperatures are below and above room temperature, respectively (*T*_C_ ≈ 260 K for La_0.6_Ca_0.4_MnO_3_ and ≈370 K for La_0.6_Sr_0.4_MnO_3_). It is expected that the mixture of La_0.6_Ca_0.4_MnO_3_ and La_0.6_Sr_0.4_MnO_3_ will present two separate phases bringing to an overlapping of the magnetic entropy change peaks. Therefore, a significant MCE will be obtained over a large range of temperatures, ranging from 260 to 370 K. Maxwell relations and Landau Theory were performed to calculate the magnetic entropy change (Δ*S*_M_). From the field dependence of the isothermal entropy change data, critical exponents were determined. From the magnetic entropy change (Δ*S*_M_*vs. M*^2^), spontaneous magnetization (*M*_spont_) was obtained and then compared to that extracted from the classical extrapolation of the Arrott curves (*μ*_0_*H*/*M vs. M*^2^).

## 2. Experiment

### 2.1. Synthesis

The 0.75La_0.6_Ca_0.4_MnO_3_/0.25La_0.6_Sr_0.4_MnO_3_ composition (SC. 3-2) was prepared by two steps. First, the two mother samples La_0.6_Ca_0.4_MnO_3_ (S0C1) and La_0.6_Sr_0.4_MnO_3_ (S1C0) were prepared *via* citric-gel method using nitrate reagents: La(NO_3_)6H_2_O, Ca(NO_3_)_2_4H_2_O, Mn(NO_3_)_2_6H_2_O and Sr(NO_3_)_2_. The precursors were dissolved in distilled water. Citric acid and ethylene glycol were added to prepare a transparent stable solution. The solution was heated at 80 °C to eliminate the water excess and to obtain a viscous glassy gel. The solution on further heating at 120 °C yields a dark grayish flakes which were calcined at 700 °C for 12 h. Then, the powder was pressed into pellets and finally sintered at 900 °C for 18 h. Second, the 0.75(S0C1)/0.25(S1C0) composition was obtained by a stoïchiometric proportion of the S0C1 and S1C0 powders. Then, the mixed powder was sintered at 900 °C to obtain the desired sample (SC. 3-2).

### 2.2. Characterization

The structure and phase purity of the prepared compounds were checked by powder X-ray diffraction technique with CuKα radiation (*λ* = 1.5406 Å), at room temperature, by a step scanning of 0.015° in the range of 20° ≤ 2*θ* ≤ 80°. The morphologies of the surfaces of all samples were examined by scanning electron microscopy (SEM). This technique was used also to prepare a histogram of grain size. The magnetization curves *versus* temperature were performed under an applied magnetic field of 0.05 T with a temperature ranging from 5 to 450 K. Isothermal magnetization data as a function of magnetic field were obtained with dc magnetic fields from 0 to 5 T.

## 3. Results and discussion

### 3.1. Structural properties

**Fig. 1:**
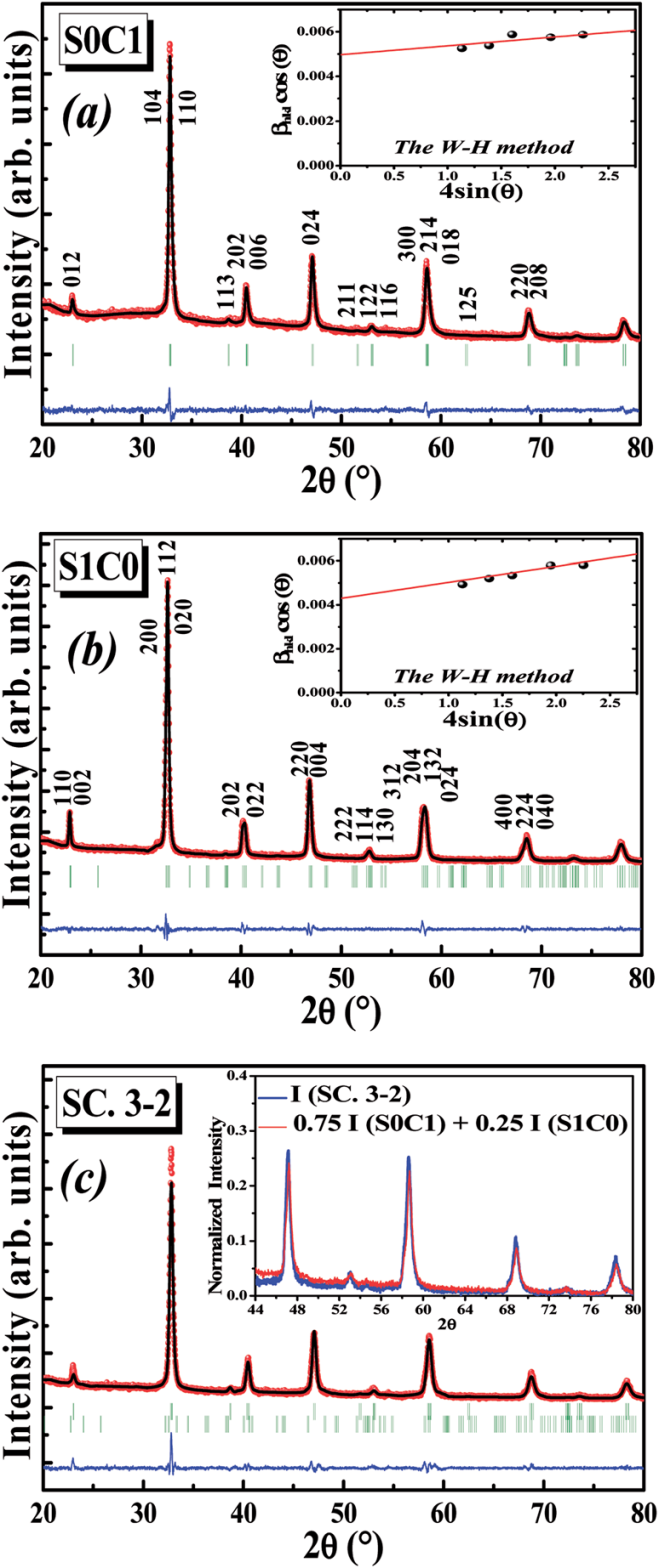
Rietveld refinement of S0C1, S1C0 and SC. 3-2 compounds. Observed (solid circles) and calculated (solid line) XRD patterns are compared (blue line). Bragg reflections are indicated by ticks. The insets show the Williamson–Hall plot of S0C1 and S1C0 compounds and the comparison between the diffraction intensity of SC. 3-2 compound and the calculated one.

Fig. 1a and b display the Rietveld refinement of the X-ray diffraction patterns of S0C1 and S1C0 compounds, respectively, analyzed using Fullprof program.^41^ The fitting between the observed and calculated spectra is relatively good. All the diffractograms are fine and intense without any detectable foreign phase confirming the good crystallization of materials. S0C1 is indexed in the rhombohedral structure with *R*3̄*c* space group. Whereas, S1C0 adopts the orthorhombic structure with *Pbnm* space group. Detailed results of Rietveld refinements are summarized in Table 1. The average crystallite size is calculated using the Williamson–Hall method. According to this approach, the X-ray line broadening is the sum of the contribution from crystallite size (*β*_size_) and the broadening caused by the lattice strain (*β*_strain_) present in the material,^42^*i.e.*:

1 *β* = *β*_size_ + *β*_strain_

where

**Table 1:** Refined structural parameters for S0C1, S1C0 and SC. 3-2 compounds

Compound	S0C1	S1C0	SC. 3-2	
Space group	*R*3̄*c*	*Pbnm*	Phase1: *R*3̄*c* (75.81%)	Phase2: *Pbnm* (24.19%)
**Cell parameters**				
*a* (Å)	5.457 (1)	5.507 (1)	5.455 (2)	5.473 (1)
*b* (Å)	5.457 (1)	5.461 (1)	5.455 (2)	5.543 (9)
*c* (Å)	13.371 (2)	7.733 (1)	13.399 (6)	7.425 (2)
V/FU (Å^3^)	57.131	58.140	57.558	56.325

**Atoms**				
La, Ca, Sr site (*x*, *y*, *z*)	0.00000	1.00004 (16)	0.00000	0.9977 (5)
	0.00000	0.00422 (18)	0.00000	0.0137 (6)
	0.75000	0.25000	0.75000	0.25000
Mn site (*x*, *y*, *z*)	0.00000	0.50000	0.00000	0.50000
	0.00000	0.00000	0.00000	0.00000
	0.00000	0.00000	0.00000	0.00000
O_1_ site (*x*, *y*, *z*)	0.45015 (2)	0.05709 (4)	0.4393 (3)	0.0394 (9)
	0.00000	0.50189 (12)	0.00000	0.5042 (9)
	0.25000	0.25000	0.25000	0.25000
O_2_ site (*x*, *y*, *z*)		0.77455 (4)		0.7327 (8)
		0.23601 (7)		0.2675 (3)
		0.01565 (4)		0.0350 (0)

**Bond angles and bond lengths**				
*θ*_Mn−O1−Mn_ (°)	163.9	157.54	160.54	166.64
*θ*_Mn–O2–Mn_ (°)		160.5		162.88
*d*_dMn–O1_ (Å)	1.9487	1.96	1.9587	1.869
*d*_dMn–O2_ (Å)		1.99		1.9724
		1.959		1.9666

**Agreement factors**				
*R*_F_ (%)	1.34	1.28	2.85	13.4
*R*_B_ (%)	1.7	1.26	4.84	16.2
*R*_p_ (%)	20.8	15.4	20.2	
*R*_wp_ (%)	14.3	11.3	15.2	
*R*_exp_ (%)	11.18	8.29	10.09	
*χ* ^2^ (%)	1.733	1.911	2.27	

• 
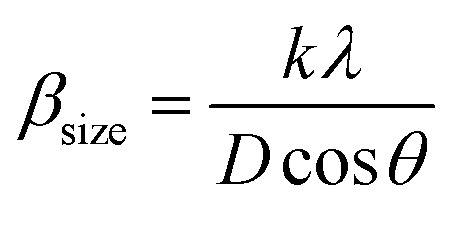
, with *λ* = 1.5406 Å is the wavelength of Cu Kα radiation, *K* = 0.9 is the shape factor, *β* is the full-width at half-maximum of an XRD peak in radians and *θ* is the Bragg angle.

• *β*_strain_ = 4*ε* tan *θ*, with *ε* is a coefficient related to strain effect on the crystallites.

The instrument broadening effect was eliminated by subtracting the value of the full width at half maximum (*β*_i_) from (*β*_size_) of a standard sample such as silicon.^43^

So, eqn (1) becomes:2
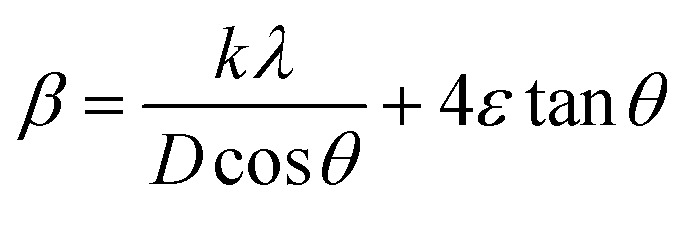
3
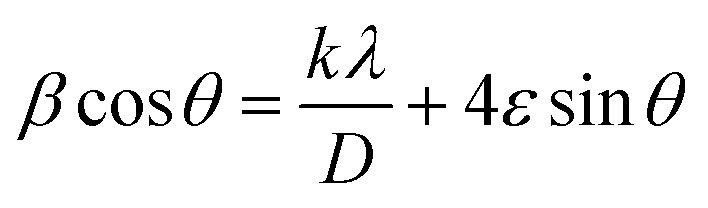


The plot of (*β* cos *θ*) (axis-*y*) as a function of (4 sin *θ*) (axis-*x*) corresponding to the strongest peaks of S0C1 and S1C0 is shown in the inset of Fig. 1a and b, respectively. Hence, by plotting *β* cos *θ vs.* 4 sin *θ*, the crystallite size *D*_W–H_ and the microstrain *ε* are achieved from the *y*-intercept and the slope of the linearly fitted data, respectively. The values of (*D*_W–H_ and *ε*) are found to be (27 nm and 0.0004) for S0C1 and (32 nm and 0.0007) for S1C0.

After the formation of S0C1 and S1C0, the two mother compounds, the SC. 3-2 composite has been successfully prepared with (0.75(S0C1)/0.25(S1C0)) composition. The XRD pattern of composite under investigation is presented in Fig. 1c. The refinement results are listed in Table 1. No phases other than S0C1 and S1C0 were detected. We note the coexistence of the rhombohedral and orthorhombic structures with percentages nearby to those introduced at the beginning; 75 and 25%, respectively. To further corroborate our result, the XRD intensity of SC. 3-2 composite is compared to the sum of those of S0C1 and S1C0 compounds multiplied by 0.75 and 0.25 mole fractions, respectively. These intensities are found to be equal (inset of Fig. 1c).

**Fig. 2:**
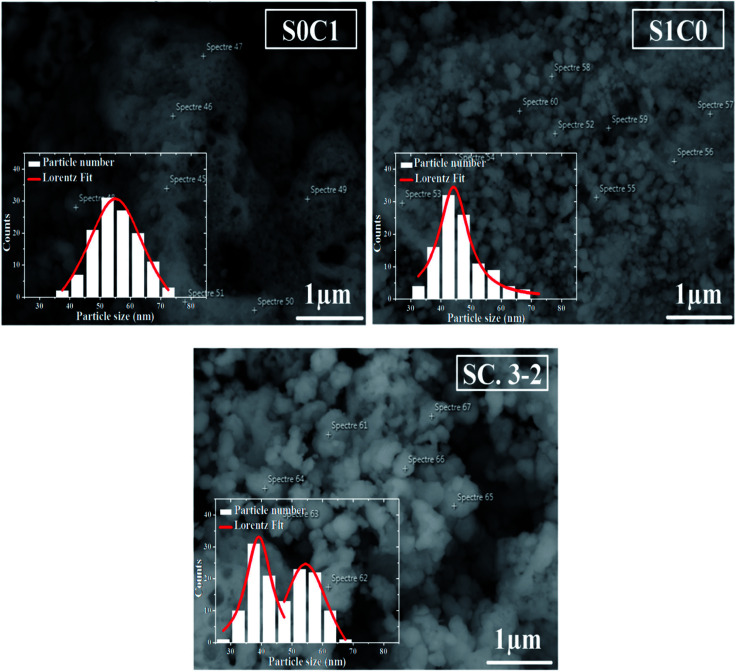
SEM image of S0C1, S1C0 and SC. 3-2 compounds. The inset shows the size distribution histogram.

SEM micrographs and grains size distribution fitted by using a Lorentzian function are depicted in Fig. 2 for all compounds. S0C1 grains are bigger than those of S1C0. Their average size is estimated to be respectively 55 and 44 nm. It is worth noting the smaller value of the XRD crystallite size compared to the SEM grain size which proposes crystallites collectivization inside the grain.^44^ SEM micrograph of SC. 3-2 shows that larger S0C1 grains are well segregated by smaller S1C0 grains signifying that the proposed composite is the mixture of two phases. Similar results have been reported by Chang *et al.*^45^ The size distribution histogram of SC. 3-2 reveals the presence of two distinctive grain size distributions of about 40 and 55 nm which are absolutely related to S1C0 and S0C1 compounds, respectively.

### 3.2. Magnetic properties

**Fig. 3:**
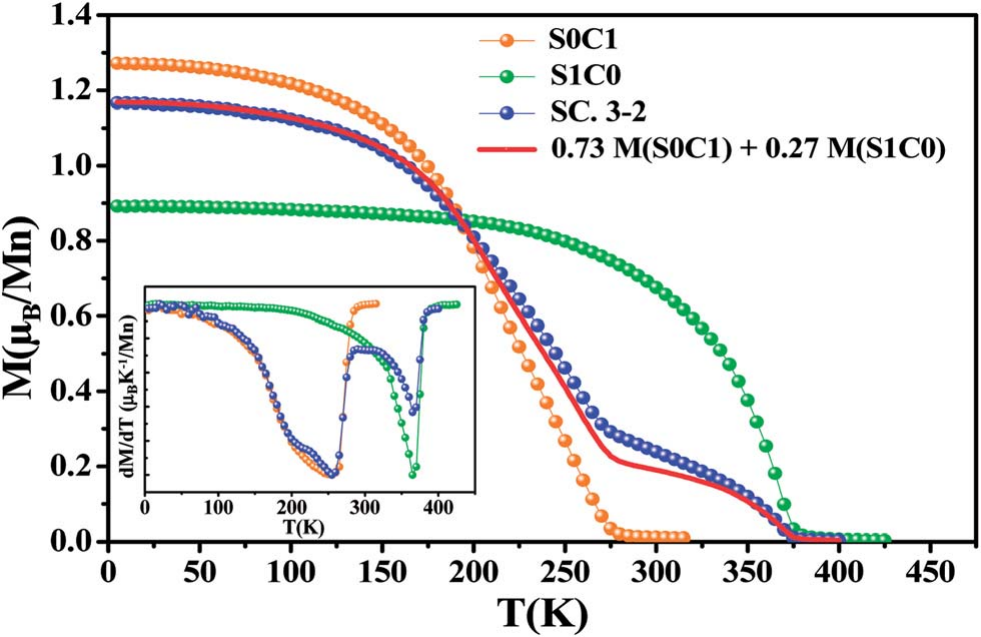
The temperature dependence of the magnetization measured under a magnetic field of 0.05 T for S0C1, S1C0 and SC. 3-2. The solid line represents the calculated magnetization *versus* temperature for SC. 3-2 compound given by the eqn (4) at *μ*_0_*H* = 0.05 T. The inset exhibits the plot of d*M*/d*T* curve as a function of temperature.

Magnetic measurements as a function of temperature for all compounds performed under an applied magnetic field of 0.05 T are introduced in Fig. 3. With decreasing temperature, the two mother compounds S0C1 and S1C0 exhibit a single magnetic phase transition from paramagnetic (PM) to ferromagnetic (FM) state at Curie temperature *T*_C_. The later, defined as the temperature corresponding to the minimum of first order derivative of the magnetization (d*M*/d*T*) *vs. T*, corresponds to 255 K for S0C1 and 365 K for S1C0 (inset of Fig. 3). These values are in agreement with those reported in references.^46,47^ As the Curie temperature values of S0C1 and S1C0 are below and above room temperature, respectively, these samples can be exploited in a wide temperature range containing room temperature. Therefore, we just should mix them to obtain a composite material usable in magnetic refrigeration technology.^48^

For SC. 3-2 composite, the *M*(*T*) curve undergoes two distinctive magnetic phase transitions at about 255 and 365 K related to S0C1 and S1C0 compounds, respectively, which is consistent with the structural observations. Our result is confirmed by using a numerical method expressed as a rule of mixtures sum:^49,50^

4 *M*(SC. 3-2) = 0.73 *M*(S0C1) + 0.27 *M*(S1C0)

where (0.73, 0.27) are the corresponding weight fractions to mole fractions (0.75, 0.25) of mother compounds (S0C1, S1C0).

A good agreement is noticed between the experimental curve and the theoretical one (Fig. 3). Similar behavior has been observed in the polycrystalline La_0.8_Sr_0.2_MnO_3_/La_0.7_Ca_0.3_MnO_3_ composite^51^ with *T*_C_ equal to 205 and 306 K for La_0.7_Ca_0.3_MnO_3_ and La_0.8_Sr_0.2_MnO_3_ compounds, respectively.

From the *M*(*T*) curves, it is worthy to note that the magnetization *M* (0.05 T, 5 K) value of S0C1 compound is higher than that of S1C0 one. This apparent difference can be due to the effect of the variance of the A-cation radius distribution noted *σ*^2^, formed by both the variance in the distribution of Mn–O and A–O distances; *σ*^2^(Mn–O) and *σ*^2^(A–O), respectively.^52^ The *σ*^2^(Mn–O) quantifies the orthorhombic distortion and *σ*^2^(A–O) quantifies the local one. An orthorhombic distortion induces the localization of the carriers, and therefore leads to the reduction of the ferromagnetic behavior and increase of the super-exchange antiferromagnetic interactions. In fact, for S1C0 compound, crystallized in the orthorhombic structure, the contribution of both orthorhombic and local distortions is evident, inducing a clear decrease of the ferromagnetic interactions, and thus a reduction of the magnetization. However, for S0C1 compound, the structural transition to the rhombohedral phase is characterized by a suppression of the orthorhombic distortion *σ*^2^(Mn–O), but the local distortion *σ*^2^(A–O) continues to exist. The suppression of the orthorhombic distortion is accompanied by a delocalization of carriers between the Mn ions, leading to an enhancement of the ferromagnetic behavior, which can explain the clear increase of the magnetization for S0C1 compound. As the rhombohedral structure is dominant for SC. 3-2 compound (∼75.81%), a similitude of the magnetization behaviors and values at low temperature with the S0C1 one can be noted. The observed little difference between the M (0.05 T, 5 K) of S0C1 compound and that of SC. 3-2 one can be related to the secondary orthorhombic phase (∼24.19%) existing in SC. 3-2.

**Fig. 4:**
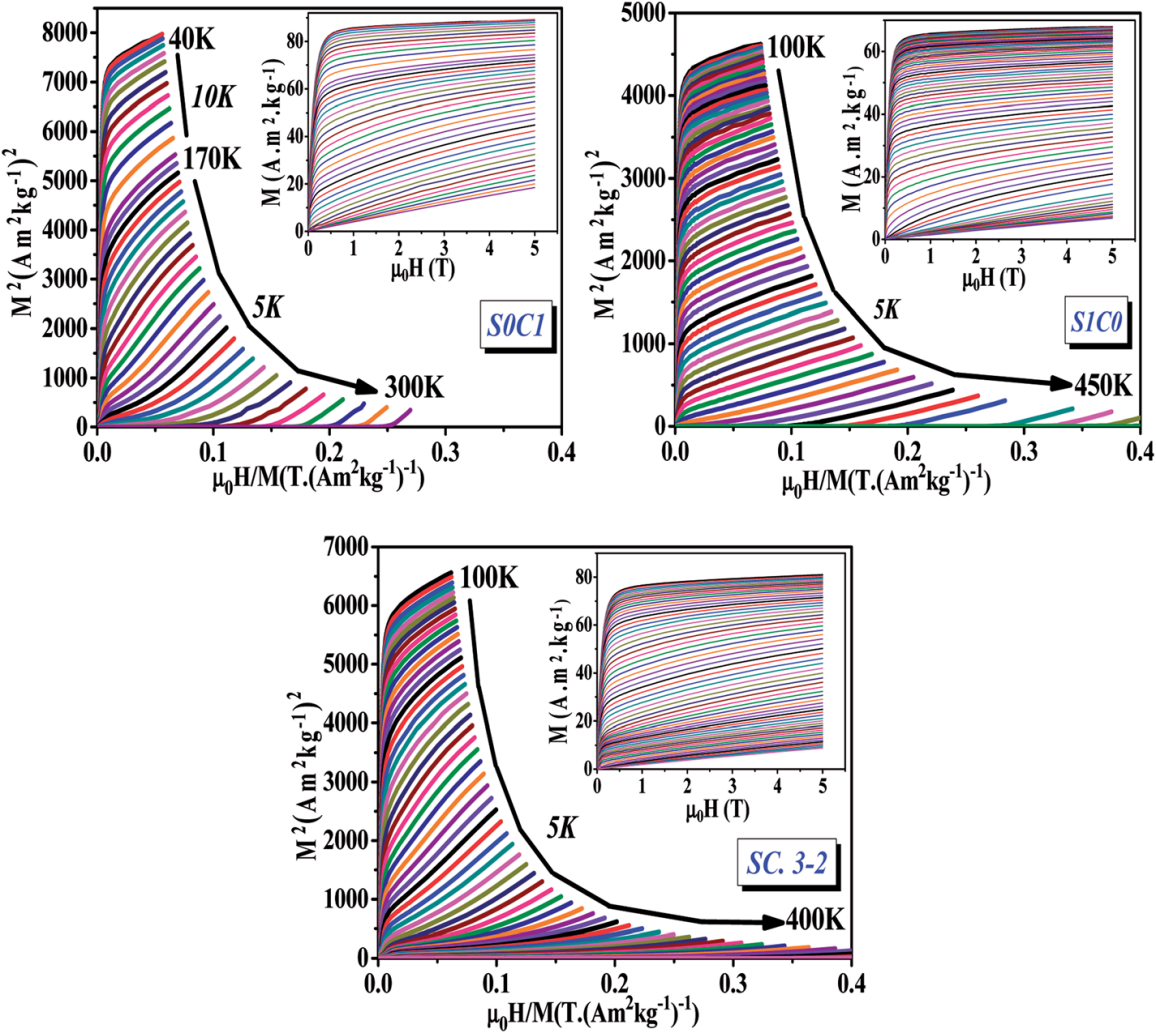
Arrott plots (*M*^2^*vs. μ*_0_*H*/*M*) for S0C1, S1C0 and SC. 3-2 compounds measured at different temperatures. The inset displays the isothermal magnetization curves.

Isothermal magnetizations *versus* applied magnetic field *M*(*μ*_0_*H*, *T*) for all compounds measured at various temperatures are illustrated in the inset of Fig. 4. At low temperature values, the *M*(*μ*_0_*H*, *T*) data increases sharply at low magnetic field and then saturates as field value increases which is a feature of FM material. At high temperature values, magnetization changes linearly with the applied magnetic field as is typical for PM material.

The main panel of Fig. 4 shows the Arrott plots of (*M*^2^*vs. μ*_0_*H*/*M*) which are derived from the isothermal magnetizations. According to the criterion suggested by Banerjee,^53^ the order of the magnetic phase transition can be verified from the sign of the slope of Arrott curves (*M*^2^*vs. μ*_0_*H*/*M*). The positive slope observed for all studied temperatures reveals that the magnetic transition between the FM and PM phase is of the second order which is highly desired in the sense of magnetic refrigeration applications.^54^

### 3.3. Magnetocaloric properties

In order to assess the efficiency of our compounds in the magnetic refrigeration systems, the magnetic entropy change(Δ*S*_M_), which is associated with the magnetocaloric effect, is determined indirectly from the isothermal magnetization curves using the approximated Maxwell equation:^55^5
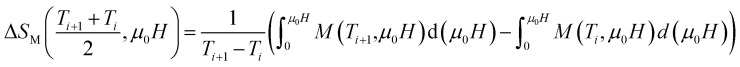
where *M*_*i*+1_ and *M*_*i*_ are the experimental magnetization values measured at *T*_*i*+1_ and *T*_*i*_ temperatures in magnetic field Δ*H*.

**Fig. 5:**
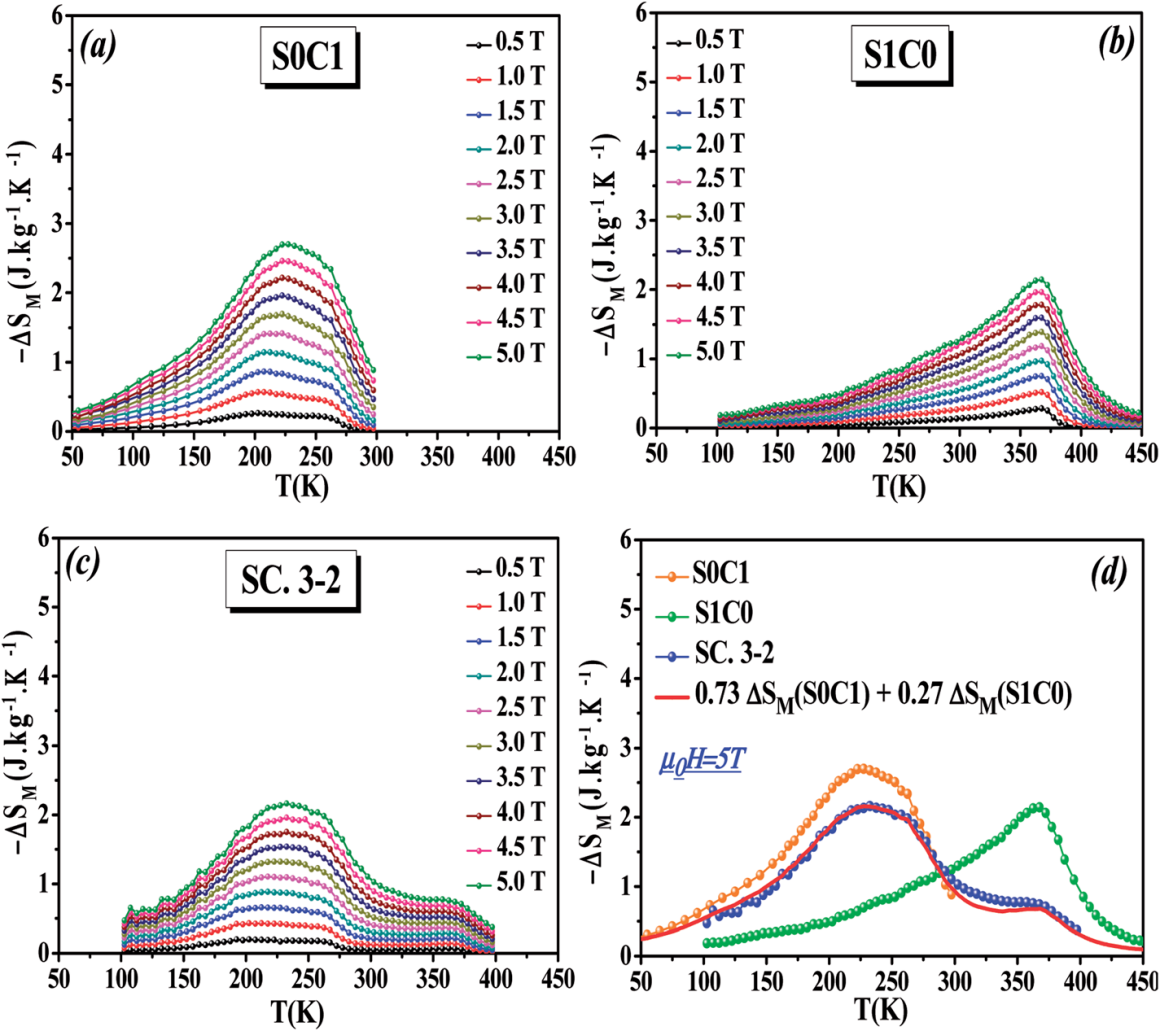
Magnetic entropy change (−Δ*S*_M_) as a function of temperature at various magnetic fields between 0.5 and 5 T for (a) S0C1, (b) S1C0 and (c) SC. 3-2 compounds. (d) Magnetic entropy change (−Δ*S*_M_) as a function of temperature for S0C1, S1C0 and SC. 3-2 compounds at *μ*_0_*H* = 5 T.

The temperature dependence of the magnetic entropy change (−Δ*S*_M_(*T*)) of the studied specimens are calculated at various external magnetic fields and plotted in Fig. 5a–c. As can be seen that the magnitude of (Δ*S*_M_) increases with magnetic field increasing which is indicative of much larger entropy change to be expected at higher magnetic field.

To easy compare, Fig. 5d displays the magnetic entropy change *versus* temperature for S0C1, S1C0, and SC. 3-2 compounds under an applied magnetic field change of 5 T. The magnitude of (Δ*S*_M_) reaches its maximum near the transition temperature with a value of 2.69 J kg^−1^ K^−1^ at 255 K for S0C1 and 2.14 J kg^−1^ K^−1^ at 365 K for S1C0. The (Δ*S*_M_) peak of S0C1 seems broader than that of S1C0. Such a broadening of (Δ*S*_M_) peak may be related to the increasing of surface/volume ratio; the smaller the crystallite size, the larger the proportion of the surface, whose FM coupling is weaker than that of the core. This could give a dispersion of Curie temperature, and therefore a wide magnetic transition.^56,57^ Similar results have been reported by Andrade *et al.*^58^ As expected for SC. 3-2, the magnetic entropy change curve exhibits double peaks which correspond to the (Δ*S*_M_) peaks of S0C1 and S1C0. In order to reinforce our findings, the (−Δ*S*_M_(*T*)) curve of SC. 3-2 is estimated theoretically using the following rule of mixture:

6 Δ*S*_M_(SC. 3-2) = 0.73 Δ*S*_M_(S0C1) + 0.27 Δ*S*_M_(S1C0)

A good agreement is noted between the two curves (Fig. 5d). It is worth highlighting that the (Δ*S*_M_) peak is dominated by the component with higher concentration.^48^ The most noticeable criterion of the composite system under investigation is the broadening of the magnetic entropy change compared to those of the individual components, leading to an enhancement of the full width at half maximum (δ*T*_FWHM_) of the magnetic entropy change curve.

For magnetic materials with second order phase transition, the field dependence of the magnetic entropy change can be approximated by a universal law of the field:^59^

7 Δ*S*_M_ ∝ (*μ*_0_*H*)^*n*^

where 
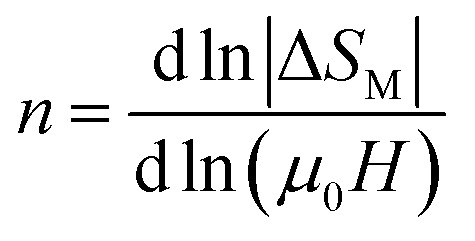
 is assigned to a parameter characteristic of magnetic ordering.^59,60^

In the case of *T* = *T*_C_, the exponent *n* becomes an independent field:^61^8
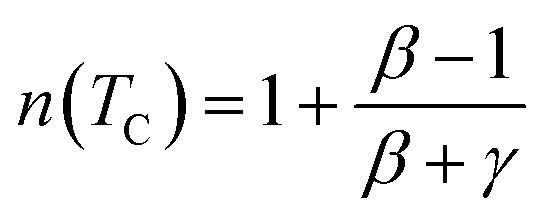
where *β* and *γ* are the critical exponents.

Using the Widom relation^62^
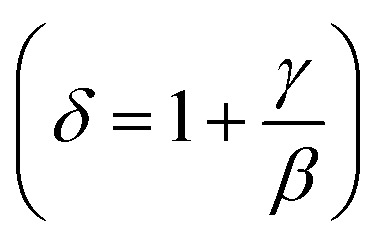
, eqn (8) can be expressed as:9
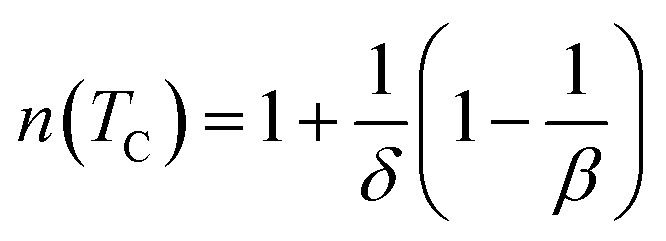


**Fig. 6:**
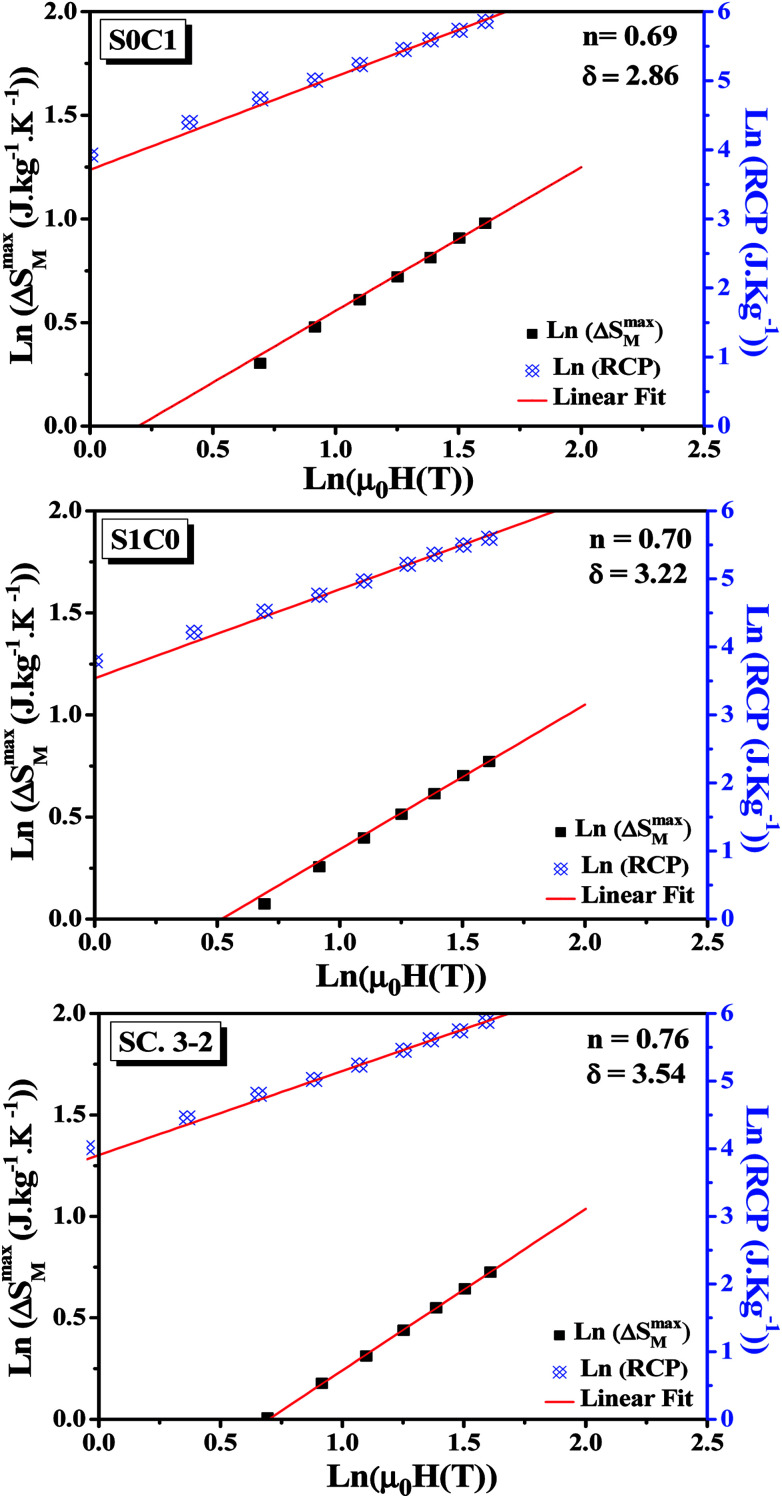
Variation of (ln(Δ*S*^max^_M_) *vs.* ln(*μ*_0_*H*)) and (ln(RCP) *vs.* ln(*μ*_0_*H*)) for S0C1, S1C0 and SC. 3-2 compounds.

By fitting the data of Δ*S*_M_*vs. μ*_0_*H* on the ln–ln scale (Fig. 6), the obtained values of the exponent *n* are 0.69, 0.70 and 0.76 for S0C1, S1C0, SC. 3-2, respectively, which show a slight discrepancy with the predicted value of 2/3 in the mean field approach.^63^ The deviation of *n* value from the mean field behavior can be explained by the presence of magnetic inhomogeneities in the vicinity of the transition temperature.^64^

It should be noted that the magnetic entropy change is not the only parameter deciding about the usefulness of materials. There is a need for materials which can transport heat at relatively large temperature difference between the cold and the hot sinks in the ideal refrigeration cycle. This feature is accounted by the magnitude (−Δ*S*^max^_M_) and the full width at half maximum (δ*T*_FWHM_) of the magnetic entropy change curve. Therefore, the amount of transferred heat may be described by the so called relative cooling power (RCP)^58^ defined by the following formula:

10 RCP = (−Δ*S*^max^_M_) × δ*T*_FWHM_

At *μ*_0_*H* = 5 *T*, the RCP reaches the value of 338, 264 and 360 J kg^−1^ for S0C1, S1C0 and SC. 3-2, respectively. It is clearly apparent that a large δ*T*_FWHM_ gives rise to a large RCP value. Therefore, the improvement of RCP values is more likely referring to the improvement of δ*T*_FWHM_ rather than (−Δ*S*^max^_M_). Comparable findings were reported by Mohamed *et al.*^65^

To assess the applicability of our samples as magnetic refrigerants, the obtained values of the RCP in our study, compared to other magnetic materials,^51,66–72^ are summarized in Table 2.

**Table 2:** Summary of magnetocaloric properties of S0C1, S1C0 and SC. 3-2 compared to other magnetic materials

Compound	*μ*_0_ *H*(*T*)	*T*_C_ (K)	(−Δ*S*^max^_M_) (J kg^−1^ K^−1^)	RCP (J kg^−1^)	Ref.
La_0.6_Ca_0.4_MnO_3_	2	255	1.15	140	This work
	5		2.69	338	
La_0.6_Sr_0.4_MnO_3_	2	365	0.97	98	
	5		2.14	264	
0.75La_0.6_Ca_0.4_MnO_3_/0.25La_0.6_Sr_0.4_MnO_3_	2	—	0.88	142	
	5		2.16	360	
Gd	2	294	5.5	164	66
	5		10.2	410	67
La_0.7_(Ba, Sr)_0.3_MnO_3_	2	316	1.27	75.74	68
La_0.8_K_0.2_MnO_3_	5	281	3.71	160	51
La_0.67_Ba_0.33_MnO_3_	5	292	1.48	161	69
La_0.75_Sr_0.25_Mn_0.8_Cr_0.2_O_3_	5	278	3.85	323	70
0.52La_2/3_Ba_1/3_MnO_2.98_/0.48La_2/3_Ba_1/3_MnO_2.95_	1	—	1.73	66.4	71
0.7Pr_0.6_Sr_0.4_MnO_3_/0.3BaTiO_3_	2	273	1.86	38.31	72
La_0.8_Ca_0.2_MnO_3_/La_0.8_K_0.2_MnO_3_	5	—	3.10	217	51

The field dependence of RCP for our samples is also analyzed. It can be expressed as a power law:^64^11
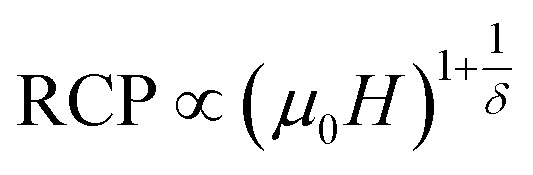
where *δ* is the critical exponent of the magnetic transition.

The value of *δ* obtained from the fitting of RCP *vs. μ*_0_*H* plot on the ln–ln scale is found to be 2.86, 3.22 and 3.54 for S0C1, S1C0 and SC. 3-2, respectively (Fig. 6).

Using the values of *n* and *δ* according to eqn (8) and (9), the obtained values of the critical exponents *β* and *γ* are respectively 0.53 and 0.98 for S0C1, 0.51 and 1.13 for S1C0 and 0.54 and 1.37 for SC. 3-2. It is remarkable that the values of the critical exponents calculated using the magnetic entropy change are consistent with the prediction of the mean field model (*β* = 0.5, *γ* = 1, *δ* = 3).

In order to identify the participated components in the MCE around the transition temperature, we have modeled the experimental results of MCE in the frame of Landau theory,^73^ which takes into account the electron interaction and magnetoelastic coupling effects.^74^

According to the Landau theory, Gibb's free energy is expressed as:^75^12
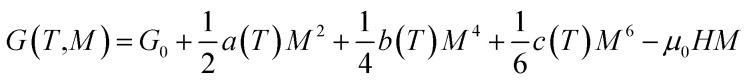
where *a*(*T*), *b*(*T*) and *c*(*T*) are temperature-dependent parameters known as Landau coefficients.

Using the equilibrium condition at *T*_C_(∂*G*/∂*M* = 0), the obtained relation between the magnetization of the material and the applied field is expressed as follows:13
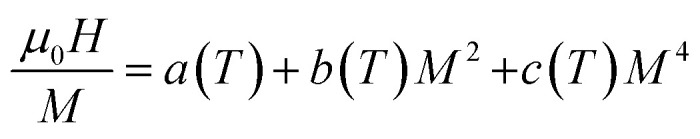


**Fig. 7:**
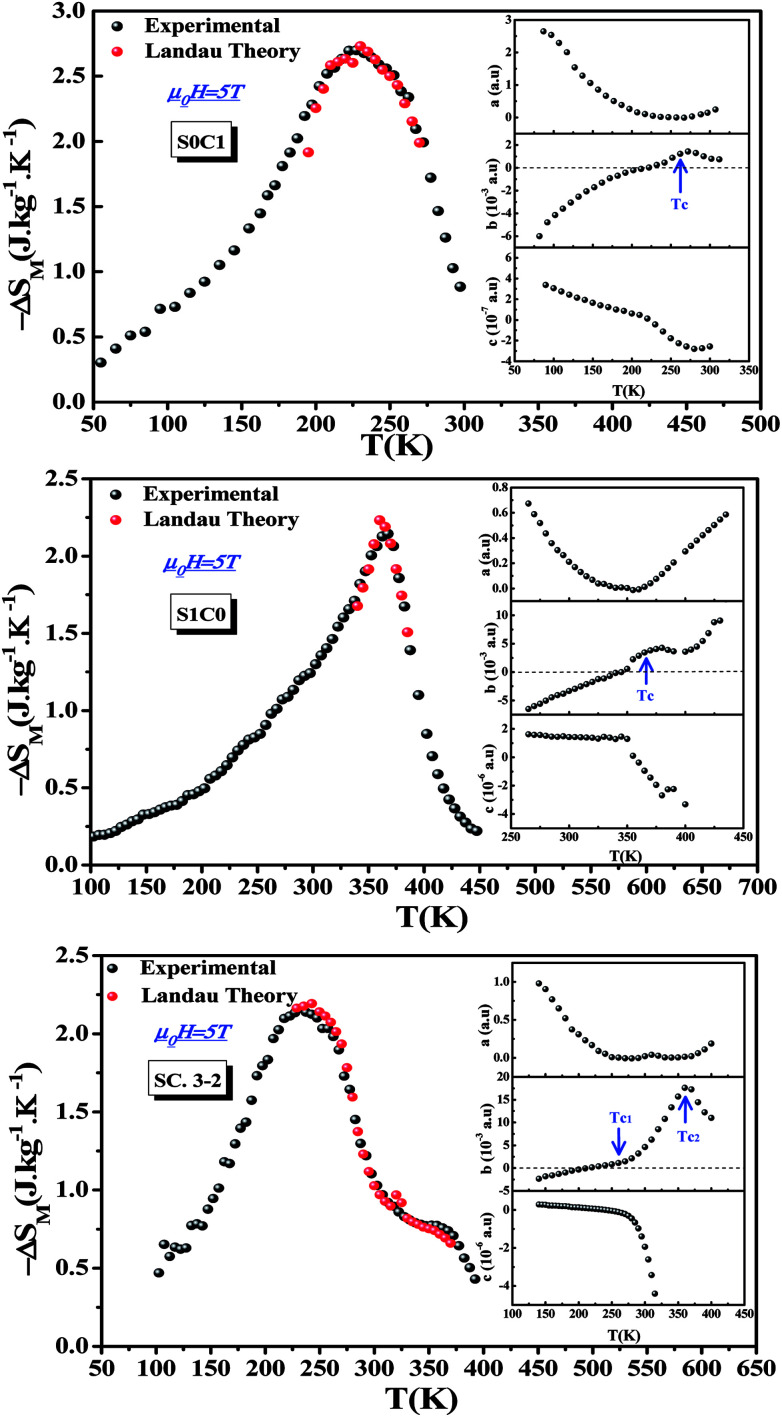
Experimental and theoretical magnetic entropy changes of S0C1, S1C0 and SC. 3-2 compounds at *μ*_0_*H* = 5 T. The inset shows the temperature dependence of Landau's coefficients.

Landau's parameters *a*(*T*), *b*(*T*) and *c*(*T*), determined from the linear region of the experimental isothermal magnetizations, are represented in the inset of Fig. 7. We can underline that the order of the magnetic phase transition is governed by the sign of Landau coefficient *b*(*T*). It can be observed that *b*(*T*) is positive at *T*_C_ for S0C1 and S1C0 and positive at *T*_C1_ (≈255 K) and *T*_C2_ (≈365 K) for SC. 3-2 which leads to conclude that all the present samples exhibit a second order magnetic transition. Similar results have been reported in previous studies.^76–80^

The magnetic entropy change is theoretically obtained from the differentiation of the free energy with respect to temperature as following:^81^14
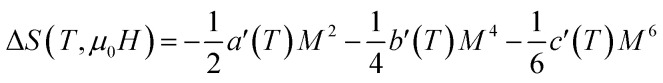
where *a*′(*T*), *b*′(*T*) and *c*′(*T*) are the temperature derivatives of the Landau coefficients.

Fig. 7 shows the magnetic entropy behavior of our samples, obtained by comparing the results coming from the Maxwell relation integration of the experimental data and the one calculated by using the Landau theory, under a magnetic field of 5 T. An acceptable concordance is observable between the experimental magnetic entropy change and the theoretical one in the vicinity of the transition temperature. The result indicates that both magnetoelastic coupling and electron interaction can explain well the MCE properties on these samples.^82^

According to the mean field theory, the magnetic entropy of magnetic materials with second order phase transition can be described as a function of magnetization as follows:^77,83,84^15
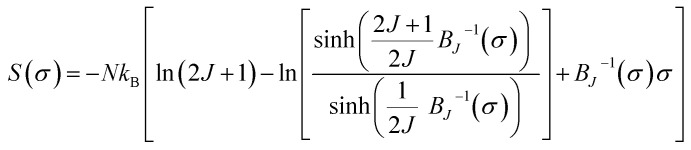
where *N* is the number of spins, *k*_B_ is the Boltzmann constant, *J* is the spin value, *B*_*J*_ is the Brillouin function for a given *J* value and *σ* = *M*/*NJgμ*_B_ is the reduced magnetization.

For small *M* values, a proportionality of magnetic entropy to *σ*^2^can be defined as:16
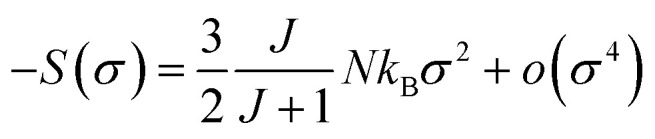


In the FM state, the system presents a spontaneous magnetization, therefore*σ* ≠ 0. Hence, considering only the first term of eqn (16), the magnetic entropy may be expressed as:17
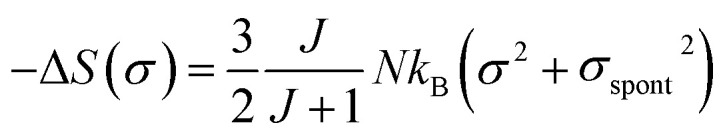


Taking the square of the reduced magnetization and substituting it for *σ*^2^ in eqn (17) results in:18
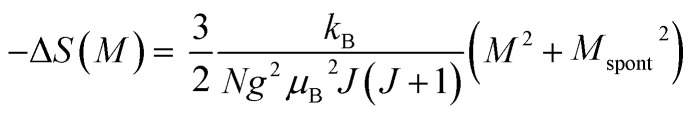
where *g* is the gyromagnetic ratio.

**Fig. 8:**
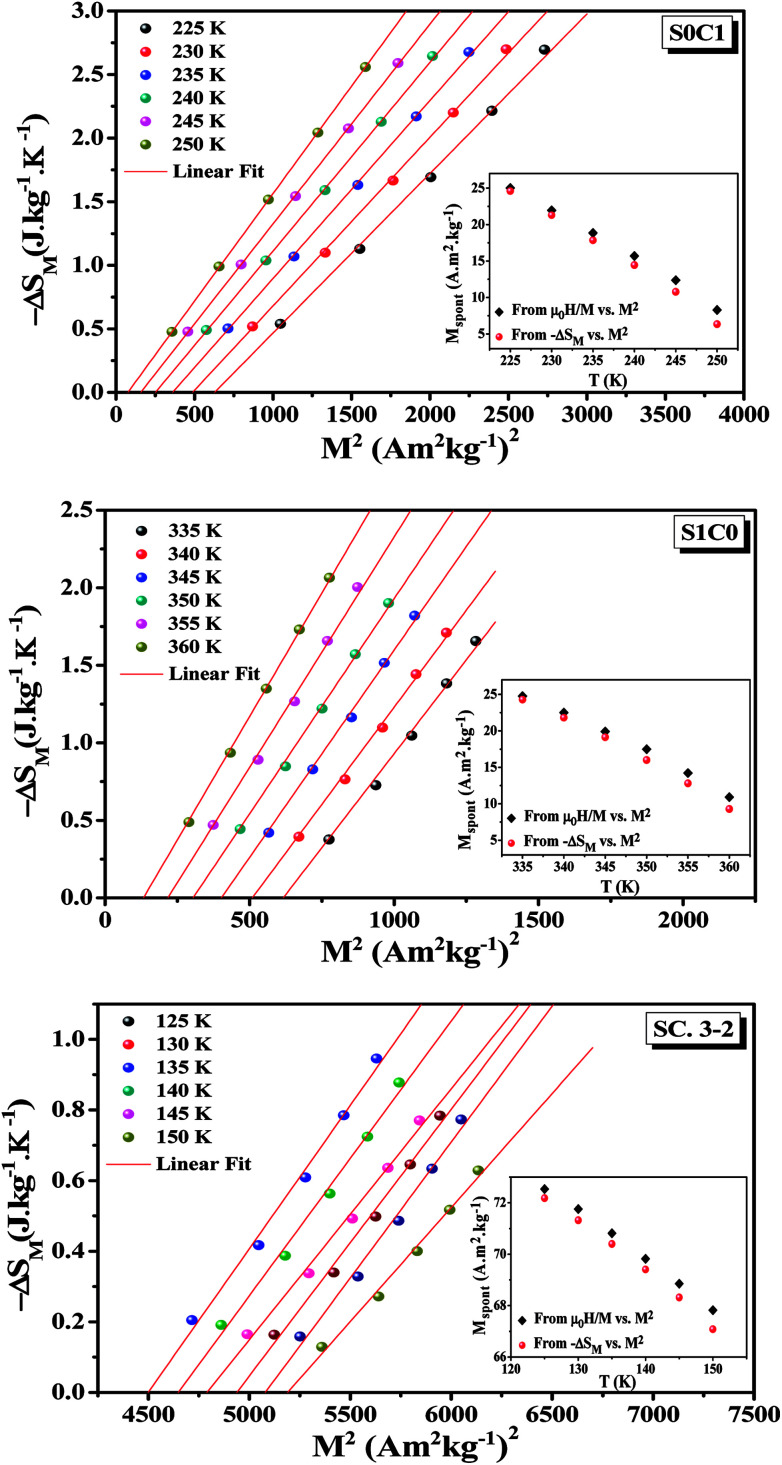
Isothermal (−Δ*S*_M_*vs. M*^2^) curves of S0C1, S1C0 and SC. 3-2 compounds. The inset displays the spontaneous magnetization deduced from the extrapolation of the isothermal (−Δ*S*_M_*vs. M*^2^) curves and from the Arrott plots (*μ*_0_*H*/*M vs.* *M*^2^).

Eqn (18) reveals that in the FM region, the isothermals (−Δ*S*_M_) *vs. M*^2^ exhibit a linear variation. By fitting the (−Δ*S*_M_) *vs. M*^2^ curves, the value of *M*_spont_ can be determined through the intersection of the straight lines with the *M*^2^axis (Fig. 8). The values of *M*_spont_, estimated from the analysis of the magnetization dependence of magnetic entropy change (Δ*S*_M_*vs. M*^2^), are compared with those deduced from the classical extrapolation of the Arrott curves (*μ*_0_*H*/*M vs. M*^2^), as shown in the inset of Fig. 8. The excellent agreement between the two methods confirms the validity of the process based on the magnetic entropy change to determine the spontaneous magnetization of S0C1 and S1C0, monophasic compounds, as well as that of SC. 3-2, biphasic compound.

## 4. Conclusion

In summary, the 0.75La_0.6_Ca_0.4_MnO_3_/0.25La_0.6_Sr_0.4_MnO_3_ (SC. 3-2) composite material has been successfully synthesized by the mixture of two citric-gel manganite-based oxides La_0.6_Ca_0.4_MnO_3_ (S0C1) and La_0.6_Sr_0.4_MnO_3_ (S1C0). The SC. 3-2 composite presents two successive second order magnetic phase transitions at 255 and 365 K related to the mother compounds S0C1 and S1C0, respectively. Consequently, a broadening of Δ*S*_M_ peak is noticed leading to a significant MCE around room temperature. SC. 3-2 exhibits an RCP value of 360 J kg^−1^ at *μ*_0_*H* = 5 T. Our findings represent a good starting point to stimulate the search for new composites with enhanced MCE properties around room temperature range. The analysis of the magnetic entropy change using the Landau theory shows an acceptable agreement with that estimated by Maxwell relations, indicating the importance of magnetoelastic coupling and electron interaction in the MCE properties of manganites system. The field dependence of the magnetic entropy change is applied to study the critical behavior. Our results are consistent with the values predicted by the mean field model. The methodology based on the analysis of the magnetic entropy change (Δ*S*_M_*vs. M*^2^) compared with the classical extrapolation of the Arrott curves (*μ*_0_*H*/*M vs. M*^2^) confirms that magnetic entropy change is a feasible method to determine the spontaneous magnetization in manganite systems.

## Conflicts of interest

There are no conflicts to declare.

## Supplementary Material
